# Distribution of Phenolic Compounds and Antioxidative Activities of Rice Kernel and Their Relationships with Agronomic Practice

**DOI:** 10.1155/2014/620171

**Published:** 2014-11-18

**Authors:** Amit Kesarwani, Po-Yuan Chiang, Shih-Shiung Chen

**Affiliations:** ^1^Department of Agriculture, School of Biosciences and Sciences, Lovely Professional University, Phagwara, Punjab 144411, India; ^2^Department of Food Science and Technology, National Chung Hsing University, Taichung 40227, Taiwan; ^3^Department of Post-Modern Agriculture, MingDao University, Changhua County 52345, Taiwan

## Abstract

The phenolic and antioxidant activity of ethanolic extract of two *Japonica* rice cultivars, Taikeng no. 16 (medium and slender grain) and Kaohsiung no. 139 (short and round grain), grown under organic and conventional farming were examined. Analyses shows that Kaohsiung no. 139 contains the highest amount of secondary metabolites and continuous farming can increase its production. Results also suggest that phenolic content under different agronomic practices, has not shown significant differences but organically grown rice has proven to be better in higher accumulation of other secondary metabolites (2,2-diphenyl-1-picrylhydrazyl (DPPH), flavonoid content, and ferrous chelating capacity). In nutshell, genetic traits and environment have significant effect on phenolic compounds and the least variation reported under agronomic practices.

## 1. Introduction

All plant-based foods have phenols, which affect their appearance, taste, odor, and oxidative stability [1]. Most of the literature, about plant phenolics, focuses mainly on fruits, vegetables, wines, and teas [2]. However, many phenolic compounds in fruits and vegetables (i.e., phenolic acids and flavonoids) are also reported in cereals. Polyphenols constitute one of the largest categories of phytochemicals as integral part of the human diet and most widely distributed in the plant kingdom as secondary metabolic products. Evidence indicates that polyphenols and flavonoids have potent antioxidant properties and free radical scavenging capabilities [3].

Different species of grains have a great deal of diversity in their germplasm resources, which can be exploited. In cereal grains, these compounds are located mainly in the pericarp and can be concentrated by decorticating the grain to produce bran, which can be incorporated into a food product (i.e., breads, cookies, and tortillas) with increased dietary fiber levels and nutraceutical properties. But the level of phenolics in plants is known to depend on cultivar and growth conditions [4]. Dietary phenolics include phenolic acid, phenolic polymers (commonly known as tannin), and flavonoids. Phenolic acids reported in cereals exist in both free and bound forms. The major phenolic acids in cereals are ferulic and p-coumaric acids. Sorghum and millet have the widest variety of phenolic acids. In rice, ferulic acid, p-coumarin acid, and caffeic acid are the major phenolic compounds and exist in the free form, the soluble conjugate form, or the insoluble bound form, which is found in dietary fiber [2].

In the adverse environmental conditions such as slow availability of nutrients to the plants under organic farming, plants become highly tolerant while providing higher antioxidant enzymes [5]. During the study of bioorganic sorghum cultivation, it was found that the enzyme activities of peroxidase and catalase have increased under organic manure and combined inoculums of N-fixing bacteria compared to control (chemically fertilized plots) [5].

Plant antioxidant levels vary according to seasons and location also, which is common phenomena within or across species and growth conditions [4, 6]. The higher antioxidative activities can be explained by differences in the phenolic concentration or other compounds in rice kernel. In spite of having nutritional advantage and beneficial effects on the health, very little is known about antioxidant properties of milled rice. Though the nutritional quality of organic food has commercialized worldwide, daily intake of secondary metabolites of milled rice grown under organic farming remains unclear. Therefore, the present study was conducted to illustrate and assess the polyphenolic compounds and antioxidant effectiveness of the milled rice under various agronomic systems.

## 2. Materials and Methods

### 2.1. Plant Materials and Preparation

The collection of two popular commercial rice cultivars, namely, Taikeng no. 16 (TK-16; medium and slender grain) and Kaohsiung no. 139 (KSH-139; short and round grain), grown under different agronomic systems (organic and conventional farming), at Taiwan had been done from 2009 to 2011. These samples were rigorously collected twice in a year as first crop and second crop seasons (harvested at June/July and November/December, resp.). The organic rice was collected from local organic farmer's market which grew under compliance with Taiwanese National Standards [7]. Conventional and organic rice samples were from the same area and grew on similar soils. This approach reduces other possible variations in safety as well as nutritional parameters. All the products were labelled with no biological or physical contaminations recorded and kept in sealed polyethylene bags under room temperature for 4 months' period.

The homogenous powdered rice was prepared by grinding and sieving under 20-mesh sieves. Such samples were packed and kept under ambient storage before further analysis conducted. All the results were expressed on a fresh weight basis.

### 2.2. Chemicals and Reagents

Ferric chloride, 3-(2-pyridyl)-5,6-bis(4-phenyl sulphonic acid)-1,2,4-triazine (Ferrozine), potassium ferricyanide, gallic acid, ascorbic acid, Folin-Ciocalteu reagent, Na_2_EDTA, 1,1-diphenyl-2-picryl-hydrazyl (DPPH), and trichloroacetic acid (TCA) were purchased from Sigma (Sigma-Aldrich GmbH, Sternheim, Germany). Similarly, other reagents and solvents used for analysis were assured of analytical and HPLC grade.

### 2.3. Preparation of Extractable Phenolic Compounds

The isolation process of phenolic compounds in rice grains is carried by blending 2 g of rice flour with 80% ethanol (25 mL). The elaborated procedure of extraction followed discussed in our previous work [8] which was slightly modified version of Adom and Liu [9] and Zhou et al. [10].

### 2.4. Determination of Total Phenolic Compounds (TPC) and Other Antioxidants

The estimation of phenolic compounds and antioxidants was following same procedure as given in our previous experiments on rice [8]. The TPC content was determined by Folin-Ciocalteu method [11] using a gallic acid standard; the TPC was calculated by a calibration curve. The results were expressed in mg of GAE (Gallic Acid Equivalent) per 100 g of white rice.

Similarly, antioxidant properties were estimated and these methods were slightly modified such as reducing power by Oyaizu [12], DPPH radical scavenging activity [13], flavonoid content [14] using quercetin as standard, and metal chelating capacity determination by Decker and Welch [15].

### 2.5. Statistical Analysis

The data were subjected to ANOVA using SAS version 8.1 (SAS Institute, Cary, NC) and expressed as means ± standard errors of each factor. Duncan's Multiple Range Test was further used to determine significant differences between means and considered statistically significant if *P* ≤ 0.05.

## 3. Results and Discussions

### 3.1. Total Phenolic Content (TPC)

Phenolic compounds of cereal grains are important for human health as they have anti-inflammatory and antiageing properties. The phenolic contents ranged from 151 to 178 mg GAE 100 g^−1^. These compounds in present study were reported to be much higher (Table 1) than previous studies of extraction either in methanol extracts [16], water extracts [17], or ethanol based on various cultivars [9, 18] but they were comparatively lower in another study of rice methanol extracts [19]. No remarkable differences in TPC have been noticed either in organic or conventional growing systems (Table 1). However, our results were in contradiction with study of Thailand rice [20], reporting higher phenolic content of rice bran in organic 1.88 mg GAE/g or postharvesting methods (product-sampling, storage, etc.) influence. It might be analytical methods or supernatant preparation that implies ready detection of these bioactive compounds in other studies [8, 21, 22]. In addition, the secondary metabolites content was reported to be much higher in rice bran [19] or pigmented rice [18–20] than light brown or white rice. The mean phenolic content of red or colored bran genotypes varies from 1.88 to 34.5 mg GAE/g in the above studies. It was noticed in our study that sample storage for certain period might bring nutritional properties deterioration as changes in metabolic activities or respiration increase cellular properties, unlike previous analysis of freshly prepared samples [8]. But, it also supports the idea of increased bioactive compounds under organic farming which is statistically equivalent to conventional rice (*P* ≤ 0.05).

**Figure 1:**
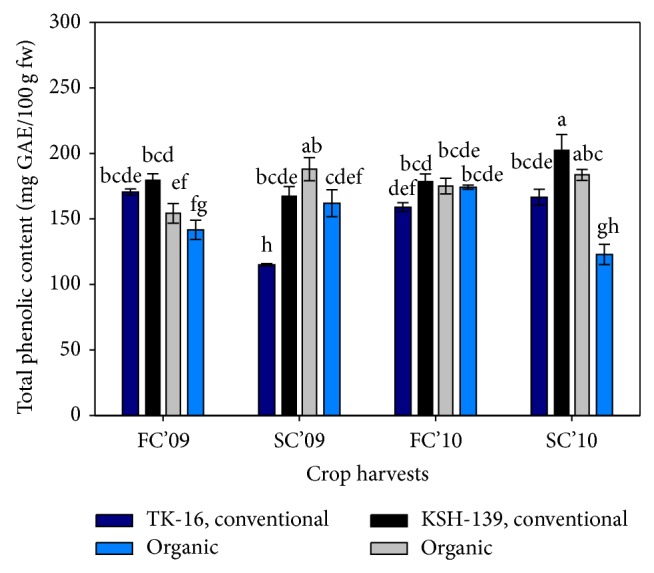
Influence of farming practices on total phenolic content (TPC) of two rice cultivars under different seasons. FC'09 = first crop (February–June) of 2009; SC'09 = second crop (August–November) of 2009; FC'10 = first crop (February–June) of 2010; SC'10 = second crop (August–November) of 2010. Values with different letters are significantly different (±S.E.) *P* ≤ 0.05 (DMRT).

Interestingly, the lowest or highest TPC was recorded (Figure 1) under conventional rice of second season crop (S4, S2). Evidence shows lower activity of phenolic component under chemical farming due to natural disruption of synthesis of antioxidants by higher accessibility of nitrogen [23] or ready availability of nutrients [24, 25]. So, plants that are grown without chemicals can increase TPC or under pathogenic pressures [26]. Similarly, other abiotic or biotic factors except nutrients availability would be detrimental in production also [6, 22, 26, 27]. Similar production of TPC in seasons (S1, S3) irrespective of agronomic practices (Table 2) is quite evident that antioxidant level remains genotypic factor or is considerably influenced by environment than farming system.

Table 2 shows that, among the genotypes, KSH-139 had effective phenolic content compared to TK-16 cultivar (Table 1) and increases periodically (Figure 1) while exact trend of TPC was noticed among both seasons also (*P* ≤ 0.05).

### 3.2. Antioxidant Activities

**Figure 2:**
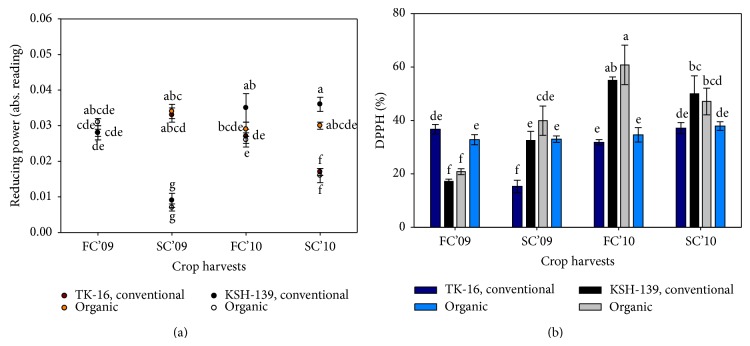
Influence of farming practices on (a) reducing power; and (b) DPPH% of two rice cultivars under different seasons. FC'09 = first crop (February–June) of 2009; SC'09 = second crop (August–November) of 2009; FC'10 = first crop (February–June) of 2010; SC'10 = second crop (August–November) of 2010. Values with different letters are significantly different (±S.E.) *P* ≤ 0.05 (DMRT).

**Figure 3:**
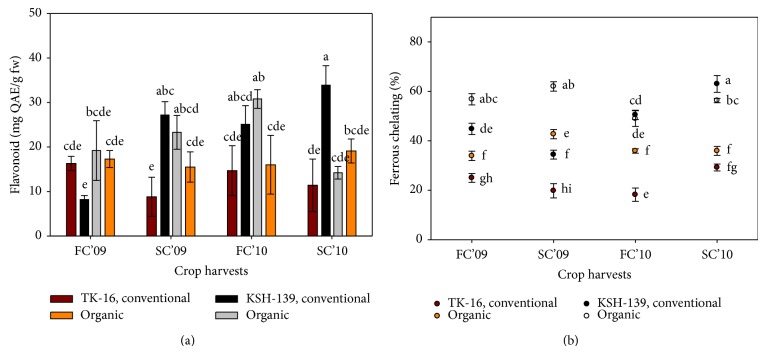
Influence of farming practices on (a) flavonoid content; and (b) ferrous chelating activity of two rice cultivars under different seasons. FC'09 = first crop (February–June) of 2009; SC'09 = second crop (August–November) of 2009; FC'10 = first crop (February–June) of 2010; SC'10 = second crop (August–November) of 2010. Values with different letters are significantly different (±S.E.) *P* ≤ 0.05 (DMRT).

Further studies of antioxidants, in these cultivars, followed similar trend of TPC results. Except ferrous chelating capacity, other antioxidant compounds show significant variation from season to season (Table 1 and Figures 2 and 3). In addition, improved and significant results (*P* ≤ 0.05) were also found in organic rice in case of DPPH and chelating activity (Tables 1 and 2). The antiradical efficiency as DPPH has been strongly correlated with pericarp colour and ranged from 10.0 to 345.3 *μ*M TE/g of rice kernel [19]. Among various combinations of factors (seasons, agronomic practices, and cultivars), it was found that cultivars or their interaction with seasons had overall significant differences than others (Table 1). In fact, antioxidant activities remain unchanged irrespective of farming system. The DPPH% was highly influenced by interaction between “season × cultivar” but in study of Goffman and Bergman [19] on analysed traits, it was indicated that interaction of seasonal differences × rice bran may not affect TPC and radical efficiency.

In the second crop (S4), conventionally grown KSH-139 cultivar had the highest reducing power, chelating (%), and flavonoid values (*P* ≤ 0.05) (Figures 2 and 3), while the overall average antioxidant activities were higher in organic cultivars during the experiment period (Table 1). The DPPH activity was reported higher 38.4% in organic rice, and similar findings were also suggested in another study of Thailand rice [20] showing stronger scavenging activity (IC_50_ 24.9 mg/mL) than conventional rice bran (IC_50_ 15.7 mg/mL). The results indicate enhancement of metabolic activities in second rice season (Figures 2 and 3) which was attributed to low metabolic rates under low temperature at early growth stages of plants and higher accumulation of these compounds but limit the rice production [28].

An inverse correlation exists between antioxidants level and higher solar radiation in summer that causes tremendous loss of these compounds [29, 30]. Among the studied genotypes, KSH-139 cultivar had excellent results (*P* ≤ 0.05) of antioxidant level with exception of reducing power which was noticed higher in TK-16 cultivar (Table 1). Also, consistent improvement was recorded in antioxidants level of KSH-139 within experimental period (Figures 2 and 3).

Overall, the results are the indication of phenolic compounds and antioxidants rely highly on genotypic as well as environmental factors [19] with minimal changes due to agronomic practices (Table 1). However, in extremities of natural condition the prevalence of antioxidant activity is also reported as organic farming.

## 4. Conclusion

The agronomic system indicated significant effect on antioxidant activities and largely under organic rice. But no changes occurred in phenolic compound under different farming system. Thus, the proponents of secondary metabolites are considered as multivariate factors which were highly influenced by season or genotypic characters. Any alteration of agronomic practices from conventional to organic farming can bring minimal or no changes in secondary metabolites but ethanolic extracts of KSH-139 cultivar under organic farming can be used as accessible source of natural antioxidants in regular diet. Thus, study was investigated on specific cultivars, so higher TPC and antioxidant isolation and identification should be encouraged using photoinsensitive rice genotypes grown under organic farming.

## Figures and Tables

**Table 1:** Phytochemicals of two rice cultivars (mean values of 2 years) as affected by treatments (conventional or organic).

Factors		Total phenolic content (mg GAE/100 g FW)	Reducing power (abs. reading)	DPPH (%)	Flavonoid (mg QAE/g fw)	Ferrous chelating (%)
Treatments	Conventional	167.4^a^	0.027^a^	34.5^b^	18.2^a^	35.6^b^
	Organic	162.7^a^	0.025^a^	38.4^a^	19.4^a^	46.6^a^

Cultivars	TK-16	151.5^b^	0.028^a^	32.4^b^	14.9^b^	30.1^b^
	KSH-139	178.6^a^	0.023^b^	40.4^a^	22.8^a^	52.1^a^

*f*-values						

	Properties	∗∗∗∗	∗∗∗∗	∗∗∗∗	∗∗	∗∗∗∗
	Season (S)	∗	∗∗∗∗	∗∗∗∗	ns	∗∗∗∗
	Treatment (T)	ns	ns	∗	ns	∗∗∗∗
	Cultivar (C)	∗∗∗∗	∗∗∗∗	∗∗∗∗	∗∗∗	∗∗∗∗
	S ∗ T	∗∗∗∗	ns	∗	ns	∗∗∗∗
	S ∗ C	∗∗∗	∗∗∗∗	∗∗∗∗	∗	∗
	T ∗ C	ns	ns	ns	ns	∗∗
	S ∗ T ∗ C	∗	ns	ns	∗∗	∗∗∗

Values for each parameter followed by a different letter within each column are significantly different, *P* ≤ 0.05 (Duncan's Multiple Range Test). Abs. = absorbance at 700 nm; GAE = Gallic Acid Equivalent; QAE = Quercetin Acid Equivalent; ns = values statistically nonsignificant (*P* > 0.05). ∗ = *P* ≤ 0.05; ∗∗ = *P* ≤ 0.01; ∗∗∗ = *P* ≤ 0.001; ∗∗∗∗ = *P* ≤ 0.0001.

**Table 2:** Phytochemicals of rice cultivars (mean values of 2 years) as affected by treatments (conventional or organic) in different seasons.

Properties	Seasons			
	S1	S2	S3	S4
Total phenolic content (mg GAE/100 g fw)	161.5^bc^	158.1^c^	171.7^a^	168.9^ab^
Reducing power (abs. reading)	0.028^a^	0.021^c^	0.029^a^	0.025^b^
DPPH (%)	26.9^b^	30.2^b^	45.6^a^	43.0^a^
Flavonoid (mg QAE/g fw)	15.3^b^	18.7^ab^	21.7^a^	19.7^ab^
Ferrous chelating (%)	40.1^b^	39.7^b^	38.4^b^	46.1^a^

Values for each parameter followed by a different letter within each row are significantly different, *P* ≤ 0.05 (Duncan's Multiple Range Test). Abs. = absorbance; GAE = Gallic Acid Equivalent; QAE = Quercetin Acid Equivalent.
